# FMRP-Driven Neuropathology in Autistic Spectrum Disorder and Alzheimer's disease: A Losing Game

**DOI:** 10.3389/fmolb.2021.699613

**Published:** 2021-10-25

**Authors:** Louis Bleuzé, Viviana Triaca, Antonella Borreca

**Affiliations:** ^1^ University de Rennes 1, Rennes, France; ^2^ Humanitas Clinical and Research Center-IRCCS, Rozzano (Mi), Italy; ^3^ Institute of Biochemistry and Cell Biology, National Research Council (CNR-IBBC), International Campus A. Buzzati Traverso, Monterotondo, Italy; ^4^ Institute of Neuroscience-National Research Council (CNR-IN), Milan, Italy

**Keywords:** FMRP, RNA binding protein, RNA metabolism, APP mRNA, Alzheimer disease, fragile X syndrome

## Abstract

Fragile X mental retardation protein (FMRP) is an RNA binding protein (RBP) whose absence is essentially associated to Fragile X Syndrome (FXS). As an RNA Binding Protein (RBP), FMRP is able to bind and recognize different RNA structures and the control of specific mRNAs is important for neuronal synaptic plasticity. Perturbations of this pathway have been associated with the autistic spectrum. One of the FMRP partners is the APP mRNA, the main protagonist of Alzheimer’s disease (AD), thereby regulating its protein level and metabolism. Therefore FMRP is associated to two neurodevelopmental and age-related degenerative conditions, respectively FXS and AD. Although these pathologies are characterized by different features, they have been reported to share a number of common molecular and cellular players. The aim of this review is to describe the double-edged sword of FMRP in autism and AD, possibly allowing the elucidation of key shared underlying mechanisms and neuronal circuits. As an RBP, FMRP is able to regulate APP expression promoting the production of amyloid *β* fragments. Indeed, FXS patients show an increase of amyloid *β* load, typical of other neurological disorders, such as AD, Down syndrome, Parkinson’s Disease, etc. Beyond APP dysmetabolism, the two neurodegenerative conditions share molecular targets, brain circuits and related cognitive deficits. In this review, we will point out the potential common neuropathological pattern which needs to be addressed and we will hopefully contribute to clarifying the complex phenotype of these two neurorological disorders, in order to pave the way for a novel, common disease-modifying therapy.

## FMRP as RNA Binding Protein: The Double Role in FXS and AD

Fragile X mental retardation protein (FMRP) is an RNA binding protein (RBP) encoded by the *fmr1* gene located on the long arm of the X chromosome. The mutation in the 5′UTR and consequent elongation of the triplet cause hypermethylation, FMRP silencing, and strong elongation of the long chromosome arm, which becomes thinner and fragile. The absence of FMRP is the main cause of a common intellectual disability, known as Fragile X Syndrome (FXS). In more detail, the main symptoms of FXS patients include cognitive disability, hyperactivity, and autism-related disorders (Santoro et al., 2012; Sethna et al., 2014). Furthermore, morphological analysis of the brain of FXS individuals reveals an increase in spine density, which is also present in the *fmr1*KO mouse model (Grossman et al., 2010). This abnormal phenotype reflects what happens locally at synapses in the absence of FMRP. Notably, FMRP has different mRNA partners relevant for synaptic plasticity. The functional role of FMRP at synapses as a regulator of the translation of these specific mRNAs is actively controlled by its phosphorylation status (Bartley et al., 2014; Bartley et al., 2016). Independently, by the increased triplet repetition in the 5′UTR of the gene, other mutations in the coding region of the *fmr1* gene have been identified. These mutations are mainly localized in the functional FMRP domain required for the RNA binding (Okray et al., 2015; Maddirevula et al., 2020) or affect its subcellular distribution (Fu et al., 2020).

Based on this primary role, most of the studies focused their attention on the local function of FMRP at synapses. However, the first report of the missense mutation c.148G>A mutation in the *fmr1* gene suggests that a subset of patients with FXS preserved the post-synaptic functions of FMRP at the KH helix alpha A domain.

The data herein demonstrate the prominent role of FMRP in RNA metabolism. Of note, several mutations have been found in the KH domain of FMRP that affect its RNA-binding site (Di Marino et al., 2014; Golden et al., 2019). Most *fmr1* mutations are associated with the overproduction of triplets in the 5′UTR of the gene. Few missense mutations in the *fmr1* gene have been well characterized and identified as responsible for the FXS phenotype (De Boulle et al., 1993, Prieto et al., 2021; Myrick et al., 2015; Sitzman et al., 2018).

For the entire list of mutations found in the *fmr1* gene, see the Table 1.

**TABLE 1:** List of missense mutation in *fmr1* gene.

Mutation in *fmr1* gene	Protein domain	References
c.11OOT>A	KH2 domain	De Boulle et al. (1993)
c.373delA	NLS	Lugenbeel et al. (1995)
g.23714GG-TA	Agenet1	Lugenbeel et al. (1995)
IVS10+14 (C > T)	KH2	Wang et al. (1997)
c.80C>A	Agenet1	Gronskov et al. (2011)
c.1444G>A	RGG	Handt et al. (2014)
c.1601G>A	RGG	Handt et al. (2014)
c.797G>A	KH1	Myrick et al. (2014)
c.377T>C	NIS	Wright et al. (2015)
c.413G>A	NLS	Myrick et al. (2015)
1457insG	RGG	Okray et al. (2015)
c.*746T>C	KH1	Suhl et al. (2015)
c.677G>A	KH1	Grozeva et al. (2015)
c.1618G>A	RGG	Hu et al. (2016)
r.1737_1738ins1737+1_?	RGG	Quartier et al. (2017)
c.990+1G>A	KH2	Quartier et al. (2017)
c.420-8A>G	NES	Quartier et al. (2017)
c.413G>A	NIS	Sitzmann et al. (2018)

**FIGURE 1:**
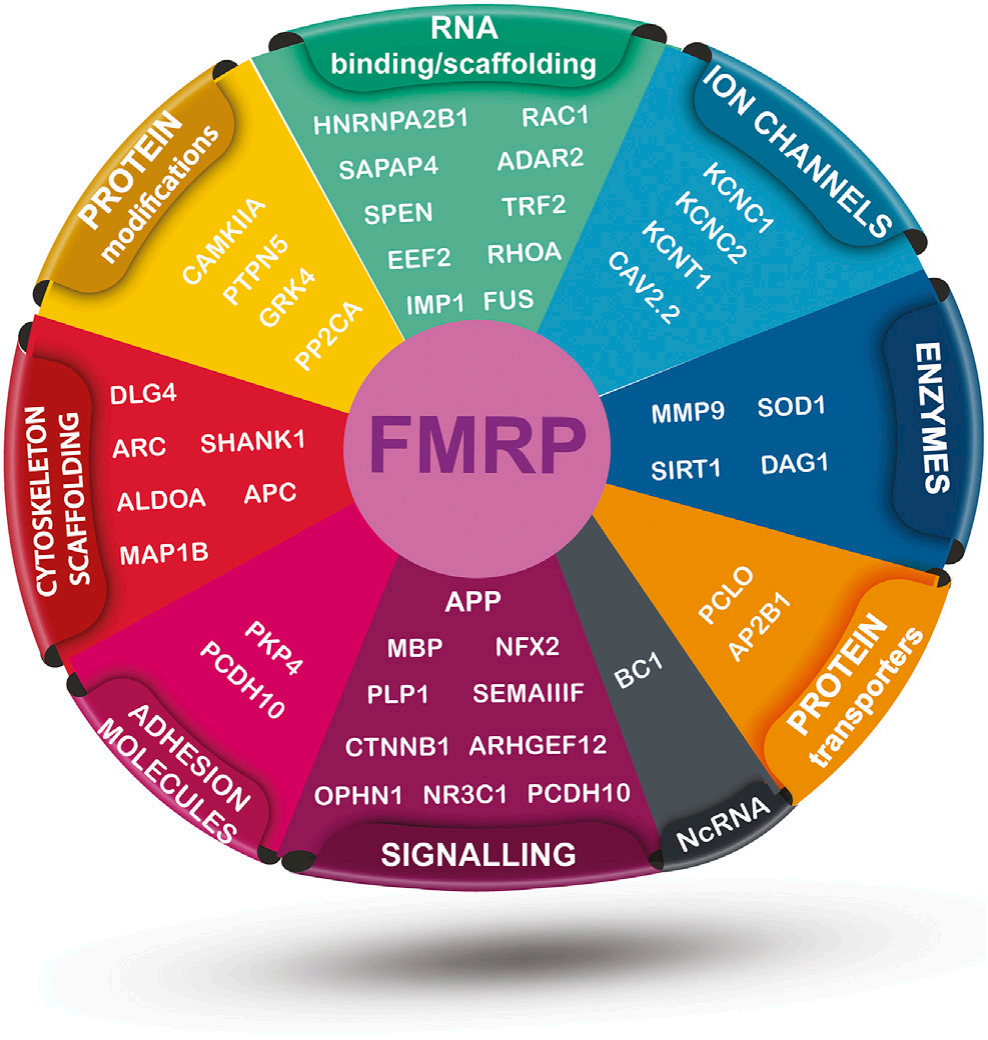
FMRP-interacting targets mRNAs sorted by their general function. The illustration includes vectors modified from “Vecteezy” and it is distributed under the Creative Commons Attribution 4.0 license (CC BY 4.0).

The main role of FMRP is related to mRNA metabolism: transport, stability and expression. In particular, FMRP is known as the RNA binding protein which is able to bind a plethora of mRNAs that have special roles in neuronal plasticity (Table 2, Figure 1). FMRP is able to bind to different mRNAs, microRNAs and long non-coding RNA (lncRNA) thanks to its peculiar structure (D’Annessa et al., 2019), including the APP mRNA (Westmark and Malter, 2007).

**TABLE 2:** FMRP mRNAs interactors.

Gene	Protein	Function	OMIM ID	References
FMR1	FMRP	RNA binding protein	309550	Santoro et al. (2011), Schaeffer et al. (2001)
APP	Amyloid precursor protein	Transmembrane protein/cell signaling	104760	Sokol et al. (2011)
MMP9	Matrix metalloproteinase 9	Enzyme/collagenase/gelatinase	120361	Janusz et al. (2013)
MAP1B	Microtubule-associated protein 1B	Microtubule-associated protein	157129	Menon et al. (2008), Dictenberg et al. (2008)
CAMK2A	Calcium/calmodulin-dependent protein kinase type II alpha chain	Protein kinase	114078	Davis et al. (2017)
DLG4	Post-synaptic density protein 95 (PSD-95)	cytoskeleton/scaffolding	602887	Santoro et al. (2012)
KCNC1	Voltage-gated potassium channel subunit Kv3.1	Transmembrane protein/ion channel	176258	Strumbos et al. (2010)
KCND2	Voltage-gated potassium channel subunit Kv4.2	Transmembrane protein/ion channel	605410	Davis et al. (2017)
ARC	Actvity-regulated cytoskeleteon-associated protein	Actin-binding protein	612461	Darnell et al. (2001)
EEF2	Eukaryotic translation elongation factor 2	GTP-binding protein	130610	Park et al. (2008)
SAPAP4	SAP 90/PSD95 associated protein	RNA-binding protein	616191	Davis et al. (2017), Brown et al. (2001), Miyashiro et al. (2003)
BC1	Brain Cytoplasmic RNA 1	ncRNA	606089	Zalfa et al. (2005), Iacoangeli et al. (2008)
GBRB	Gamma-aminobutyric acid receptor subunit BETA	Transmembrane protein receptor	137163	Wolfe et al. (2016)
MBP	Myelin basic protein	Membrane protein	159430	Giampetruzzi et al. 2013
SOD 1	Superoxide dismutase 1	Enzyme	147450	Bechara et al. (2009)
RGS5	Regulator of G protein signaling 5	GTPase activator	603276	Brown et al. (2001), Filipello et al. (2018)
RAC1	Ras-related C3 botulinum toxin substrate 1	GTP-binding protein	602048	Billuart et al. (2003)
RhoA	RHOA	GTP-binding protein	165390	Tsai et al. (2010)
ARHGEF12	Rho guanine nucleotide exchange factor 12	Cytoplasmic/membrane protein/G protein- coupled receptor binding	604763	Heulens et al. (2011)
SPEN	Msx2-interacting protein	DNA/RNA binding	613484	Brown et al. (2001)
NR3C1	Glucocorticoid receptor, group C, member 1	Membrane protein/adhesion molecule	138040	Markham et al. (2006)
CTNNB1	Catenin β-1	Membrane protein and transcriptional factor	116806	Ehyai et al. (2018)
PCLO	Piccolo	Protein transporter	604918	Pasciuto and Bagni (2014)
EEF2	Eukaryotic translation elongation factor 2	GTP-binding protein	130610	Park et al. (2008)
APC	Adenomatous polyposis coli	Microtubule-binding protein/kinase regulator	176915	Huang et al. (2015)
ALDOA	Fructose-bisphosphate aldolase A	Enzyme/scaffolding	103850	Pasciuto and Bagni (2014)
AP2B1	AP-2 complex subunit β-1	Protein transporter	601025	Van Driesche (2019)
FUS	RNA-binding protein FUS	DNA/RNA-binding protein	137070	He et al. (2017)
VDAC1	Voltage-dependent anion-selective channel protein 1	Transmembrane protein/Ion channe	604492	Chen et al. (2003)
HNRNP A2B1	Heterogeneous nuclear RiboNucleoProtein A2/B1	RNA-binding protein	600124	Sofola et al. (2007)
PKP4	Plakophilin 4	Adhesion molecule	604276	Fischer-Kešo et al. (2014)
NLGN2	Neuroligin 2	Transmembrane protein/receptor	606479	Chmielewska et al. (2019)
DAG1	Dystrophin-associated glycoprotein 1	Enzyme	613818	Pasciuto et al. (2015)
PCDH10	Protocadherin 10	Membrane protein/adhesion molecule	608286	Tsai et al. (2012)
PLP1	Myelin proteolipid protein	Membrane protein	300401	Kalinowska et al. (2015)
PTPN5	Striatum-enriched protein tyrosine phosphatase (STEP)	Phosphatase	176879	Chatterjee et al. (2018)
OPHN1	Oligophrenin 1	Membrane protein/adhesion molecule	300127	Xiao et al. (2013)
PPP2CA	Catalytic subunit of protein phosphatase 2	Phosphatase	176915	Bernard et al. (2013)
C1Q	Complement Component	Membrane Protein/complement cascade	120575	Bie et al. (2019)
A2A	A2A receptor	Transmembrane Protein/Receptor	102776	Ferrante et al. (2021)
Semaphorin 3F	Semaphorin III F	Signalling/secretion	601124	Evans et al. (2012)
Myf5a	Myogenic Factor 5	Muscle development	159990	Fujita et al. (2017)
ADAR2	Adenosine deaminase RNA-specific 2	RNA binding protein	601218	Filippini et al. (2017)
IMP1	Insulin-like growth factor 2 mRNA-binding protein 1	RNA transport factor	608288	Rackham et al. (2004)
TRF-2	Telomeric repeat binding factor 2	RNA binding protein	602027	Zhang et al. (2015)
SIRT1	Sirtuin 1	Enzyme/Chromatin silencing factor	604479	Yu et al. (2012)
Shank1	Sh3 and multiple ankyrin repeat domains 1	Scaffolding	604499	Zhang et al. (2014)
STUB1	STIP1 homologous and U box-containing protein 1	E3 ubiquitine ligase/cochaperone	607207	—
GRK4	G protein-coupled receptor kinase 4	serine/threonine kinase	137026	Maurin et al., 2015
KCNT1	Potassium channel subfamily T member 1	Transmembrane protein/ion channel	608167	Brown et al. (2010)

Theoretically, FMRP is structured in N-terminal, C-terminal and central domains. Apart its RNA binding domain, the structure of FMRP presents a nuclear export signal and a nuclear localization signal, able to transport ribonuclear complexes outside the nucleus toward synapses (Eberhart et al., 1996).

The nuclear localization signal suggests the presence of the protein in the nucleus and a related, still unknown nuclear function.

FMRP is characterized by different RNA binding motifs: namely, the KH and RGG box domains. Only a few mutations have been identified in the coding region of the *fmr1* gene (De Boulle et al.1993; Myrick et al., 2014) in FXS patients and all of them are localized in these two motifs, pinpointing their key role in protein functions (Di Marino et al., 2014). A newly reported R138Q mutation results in impaired hippocampal long-term potentiation and socio-cognitive deficits in mice (Prieto et al., 2021).

The RNA binding takes place directly or *via* non-coding RNAs, such as BC1 (Lacoux et al., 2012) or miRNAs (Ifrim et al., 2015). Interestingly, the BC1 non coding RNA is able to regulate the expression of the APP mRNA upon FMRP binding. Inhibition of BC1 or of BC1-FMRP interaction represses APP translation and hence rescues the amyloid beta level, as well as spatial learning, and memory impairments in AD mice with dysregulated RNA production (Zhang et al., 2018).

As an RBP, FMRP regulates the expression of specific mRNA locally at synapses under specific conditions and in response to external stimuli (Dolen et al., 2007). The absence of the protein causes an abnormal translational expression of its RNA partners, causing an alteration of normal synaptic plasticity. In more detail, Bear and collaborators speculated that mGluR5 serves as a sensor of ongoing synaptic excitation in the brain and, among other actions, stimulates local mRNA translation, so that the supply of rapidly turned-over proteins keeps up with demand. As with all biochemical pathways, this process is balanced by negative regulators. In the case of mGluR5-dependent protein synthesis, FMRP might be one of these negative regulators. In the absence of FMRP, one might expect that the protein synthesis-dependent effect of mGluR5 activation would be exaggerated, and indeed this is the case (Huber et al., 2002; Chuang et al., 2005).

Taken together, the findings that 1) mGluR5 stimulates protein synthesis and that 2) FMRP negatively regulates protein synthesis led to the “mGluR theory of fragile X,” which posits that the neurological and psychiatric symptoms of FXS are generated as a consequence of abnormal responses to mGluR activation (Bear et al., 2004). If this theory is valid, then it might be possible to correct some aspects of fragile X by specifically reducing mGluR5 signaling.

Under mGluR stimulation FMRP is phosphorylated and releases all partner mRNAs, thus favoring their translation. APP expression is downstream from the FMRP cascade, and patients with FXS are characterized by increase in APP level and concomitant accumulation of amyloid plaques (Westmark and Malter, 2007). In line with this, drugs aimed at inhibiting mGluR5 activation are also able to reduce Aβ accumulation in FXS patients (Westmark et al., 2009; Thomas et al., 2012; Gandhi et al., 2014). Different antagonists of mGluR5 have been shown to downregulate APP protein expression and interfere with the accumulation of amyloid-β fragments (Kumar et al., 2016).

FMRP binds the APP mRNA through the RGG box domain which recognizes a G quartet region that is extremely conserved in all species and is also present in coding region and 3′UTR of APP mRNA. The G-quartet region is also present in PSD95 mRNA, another well-known FMRP partner (Zalfa et al., 2007).

The control by FMRP of APP mRNA is confirmed by another RNA binding protein: hnRNPC (Rajagopalan et al., 1998). While FMRP acts as a repressor of APP mRNA, hnRNPC is able to stabilize it, thus facilitating APP expression (Lee et al., 2010). Notably, an imbalance has been demonstrated in both FMRP (Renoux et al., 2014a; Borreca et al., 2016) and hnRNPC expression levels in AD mouse models (Borreca et al., 2016). This alteration has been reported in the early phase of pathology, highlighting the important role of APP mRNA level upstream of any APP cleavage dysregulation. This may explain the different FMRP outcomes reported in the two studies (Renoux et al., 2014b; Borreca et al., 2016), together with the use of two different AD mouse models.

These results support a possible double role of FMRP in FXS and in AD, although further studies are required. How FMRP mutations can affect two apparently different pathologies is still unknow and the role of neuroscientists is fundamental to disclose the shared underlying mechanism.

## FMRP and Protein Synthesis in FXS and AD

The role of FMRP in protein synthesis has been clearly described. FMRP acts as translational repressor of specific target mRNAs and its absence or dysfunction affects the protein synthesis machinery.

In more detail, FMRP acts as negative regulator of local translation at synapses. By acting on RNA metabolism, FMRP is able to bind specific RNA, transfer them locally at synapses, and release them upon specific stimuli, allowing their translation when necessary.

In FXS patients, basal translation of FMRP mRNA partners is unstable and constantly active. Among them, ARC mRNA is overexpressed in FXS patients inducing AMPA internalization and unstable spine function (Park et al., 2008).

A local protein synthesis at synapses plays a relevant role in the synaptic formation and storage of the information necessary for performing specific memory tasks (Holt et al., 2019; Perrone-Capano et al., 2021).

Recent data demonstrate how protein synthesis levels are increased in individuals with FXS (Jacquemont et al., 2018; Utami et al., 2020). FXS individuals have an increase in the synthesis of *de novo* proteins such as ERK1/2 and Akt, involved in the mTOR pathway, which is known for regulating the expression of key proteins involved in neurodevelopmental and neurodegenerative diseases, such as FXS, ASD, T2D, and AD. This pinpoints its relevance for the identification of new common therapeutic strategies.

The activation of the metabotropic glutamate receptor 5 (mGluR5) in FXS triggers a cascade of reactions involving PI3K/Akt survival signal, the activation of mTOR, and its subsequent interaction with mTORC1 (Sharma et al., 2010; Casingal et al., 2020).

Following mTOR pathway activation, there is a regulation of eIF4E by 4E-BP such as CYFIP1, with increase of the protein synthesis in normal conditions (Napoli et al., 2008; De Rubeis et al., 2013). While in FXS the lack or absence of FMRP leads to an upstream dysregulation of the mTOR pathway that results in an increased protein synthesis.

Since microglia, the brain immune cells, are also involved in synaptic pruning and one of the main features of FXS individuals is excess of synapses, a general protein synthesis is fundamental for the functional role of synapses. Accordingly, alteration of protein synthesis machinery in microglia induces an autistic-like behavior (Xu et al., 2020).

Furthermore, it has been largely demonstrated the presence of FMRP in polysomes and mRNA granules in neuronal cells, suggesting its translational control in dendrites. In particular, granules are normally composed of a repertoire of mRNAs transported in a translationally silent state into dendrites for subsequent site-specific utilization at synapses undergoing protein synthesis-dependent changes (Krichevsky et al., 2001; Keene et al., 2002; Antic et al., 1998). FMRP is associated with translating polysomes and with stalled polysomes (Khandjian et al., 2004; Stefani et al., 2004; Ceman et al., 2003), confirming the idea that it gates translation after the initiation step. This means that specific mRNAs is stalled on ribosomes by FMRP, suggesting a prominent role in the first step of translational initiation.

On the other hand, AD is characterized by an accumulation of amyloid β plaques in synapses and the hyperphosphorylation of tau proteins. Several lines of evidence pinpoint that brain neurodegeneration in AD shares some common pathological patterns with FXS.

In line, FXS patients show an increase of Aβ production due to the FMRP negative control on APP mRNA. The absence of FMRP induces the release of APP mRNA which is locally translated at synapses, leading to an increase of amyloidogenic products. Amyloid β, a product of the amyloidogenic APP processing pathway, is able to bind mGluR5 and promote a positive feedback loop controlling amyloid β within normal levels in healthy conditions (Westmark, 2013). Given the key role of FMRP on controlling mGluR5 signalling and protein synthesis at synapses, an involvement of FMRP in AD typical events cannot be excluded.

Interestingly, protein synthesis alteration has been found also in AD brains (Hernandez-Ortega et al., 2016; Cefaliello et al., 2020; Ghosh et al., 2020). Polysomal profiles from wild-type (wt) and the Tg2576 AD mouse model (Borreca et al., 2020) reveals the shift of APP mRNA in the polysomal fraction, with APP more prone to be translated. Furthermore, a translational initiation factor, EIF2α is involved in AD cognitive deficit (Costa Mattioli et al., 2006). In particular, EIF2α is upregulated upon phosphorylation and the protein synthesis is blocked in late phase of AD pathology. Surprisingly, in the early phase of AD, the phosphorylation status of EIF2α is reduced, suggesting an increase of protein synthesis machinery and as a consequence a compensatory mechanism taking place in the late phase of the disease with high phosphorylation status of EIF2α and block of protein synthesis. Therefore, the block of protein synthesis will occur later in the disease, possibly as a feedback control (Borreca et al., 2020). Several protein synthesis molecules are involved in other neurodegenerative disorders such as EIF4G in Parkinson disease, thus suggesting a fundamental role of protein synthesis in the functional role of synapses and the cognitive deficit observed (Dhungel et al., 2015).

Overall, it can be hypothesized that FMRP control of mGluR5 signaling, APP expression and synaptic proteins represents a common dysregulated pathway contributing to the increase in protein expression apparently occurring in both FXS and prodromal AD.

## THE miRNA-Based Pathway in FXS and AD

Non-coding RNA plays a pivotal role in different cellular processes and are implicated in epigenetic mechanisms, translational and transcriptional processes, of interest for understanding more complicated cellular pathways.

Noteworthy, FMRP is known to be implicated in the RNA interference (RNAi) mechanism and epigenetic silencing. The process of RNAi is a very intriguing mechanism that regulates post transcriptional gene expression within the cells. The silencing of the gene is regulated by the RISC complex. In particular, AGO2 protein combines with the RISC complex before binding to the functional strand RNA, inducing silencing of gene. In this scenario, the *fmr1* gene containing full mutation CGG triplets in the 5′UTR is recognized by the RNAi complex and has a key role in the epigenetic silencing of the *fmr1* gene.

Moreover, FMRP is able to bind specifically to some miRNAs, *via* its RNA binding domain, and in particular, to two miRNAs known to be also involved in AD pathologies: miR-132 and miR-125b (Edbauer et al., 2010). The miR132 has a protective effect on neuronal cells and its expression is reduced in AD disease progression (Zhu et al., 2016). In contrast, miR-125b is extremely deleterious for neuronal cells, with an increase in the hippocampus of AD mice (Lukiw, 2007; Ma et al., 2017). Furthermore, there is ongoing debate over whether FMRP is able to bind mRNA through long non-coding RNA (lncRNA) BC1 (Zhong et al., 2010). In AD mouse models, once linked to BC1 FMRP releases the APP mRNA with full protein overexpression (Zhang et al., 2018). This idea is still controversial and understanding the molecular mechanism of APP expression is still open. BC1 is found overexpressed in AD mice models and human AD samples (Mus et al., 2007). Furthermore, inhibition of BC1 blocks APP expression in AD mouse models and rescues cognitive defects in those mice (Zhang et al., 2017). APP expression is also regulated post-transcriptionally by different miRNAs. Most of them are in the regulatory region of APP mRNA (Vilardo et al., 2010; Long et al., 2019).

A growing body of evidence indicates that miRNAs regulate synaptic homeostasis and plasticity processes, suggesting that they may be involved in early synaptic dysfunction during AD. Based on this, it is thought that miRNA may be a potential biomarker in the prodromal phase of pathology (Siedlecki-Wullich et al., 2021).

Identifying the molecular mechanisms of miRNA-driven gene expression will be helpful in understanding the complex pathologies of AD and FXS.

## Neuroinflammation Studies in FXS and AD

The brain is composed of neuronal cells, and also non-neuronal cells, such as microglia and astrocytes. The concomitant work of all brain cell is fundamental for synapses to function. The role of FMRP has been well described in neurons and synapses. The role of this protein in other brain cells has only recently been questioned. It has been demonstrated that astrocytic glutamate transport 1 (GLT1), a major glutamate transporter of glia, is reduced in *fmr1*KO astrocytes compared to wt (Higashimori et al., 2013; Higashimori et al., 2016). It has also been demonstrated that *fmr1*KO astrocytes are more active compared to wt (Cerdeno et al., 2018) and that there is a reduced number of microglia cells in *fmr1*KO mice compared to wt (Lee et al., 2019). This demonstrates the important role of different brain cell types and shows how they contribute to the physiological state of neuronal cells. In particular, astrocytes and microglia contribute to synapse refinement, and it is possible, alike that their alteration would contribute to the excessive and immature synapses observed in FXS mice models. This idea is also supported by the alteration of different cytokines in *fmr1*KO mice (Hodges et al., 2017). Furthermore, it has been demonstrated that FMRP is associated to C1q mRNA, which is fundamental for complement cascade and for the recognition of synapses to eliminate (Bie et al., 2019). In particular, artificial activation of mGluR1 signaling promotes dephosphorylation of FMRP and facilitates the local translation machinery of synaptic C1q mRNA, thus mimicking the C1q-mediated microglial phagocytosis of hippocampal glutamatergic synapses and cognitive deficiency in rodent models (Bie et al., 2019). C1q is part of the complement system and also contributes to synaptic pruning by tagging weak or damaged synapses, resulting in their subsequent phagocytosis by microglia (Schafer et al., 2012).

C1q immunoreactivity is not visualized in synapses, which indicates that C1q expression from other brain cells (microglia) might contribute to the pathological processes. In particular, this mechanism suggests a fundamental communication between neurons and microglia in physiological conditions. Alteration of C1q-mediated synaptic elimination is observed in the pathogenesis of several neurological disorders (Wang et al., 2020a; Wang et al., 2020b).

An alteration of the neuroinflammatory system is also a common feature of AD patients and it has been clearly demonstrated that the brain of AD samples are “inflamed.”

AD is a complex pathology and the involvement of non-neuronal cell types make this disease difficult to understand. The Amyloid β production and accumulation of neurofibrillary tangles extracellularly activate microglia and astrocytes (Fakhoury et al., 2018). Furthermore, it has been demonstrated how mutations in TREM2 microglia receptors are associated with Alzheimer’s disease (Ulland et al., 2018). This further suggests a prominent role of immune cells in the pathogenesis of AD. Trem2 alteration are also observed in human ASD patients (Filipello et al., 2018) suggesting a further common feature in AD and Autism.

## Amelioration of FXS Cognition and Behavior Upon Rescue of Basal Forebrain Cholinergic Neurons Deficits by AD Drugs

Neurodegeneration of cholinergic neurons of the basal forebrain (BF) and of its cortical and hippocampal targets is one of the most prominent and prototypical sign of AD pathology (Fernandez-Cabello et al., 2020; Schmitz et al., 2016). Interestingly, increasing evidence on BF abnormalities were reported in several syndromes, including Down syndrome and Rett syndrome, as well as in developmental conditions like autism spectrum disorders (ASD), in particular FXS.

Functionally, the basal forebrain cholinergic system (BFCS) has been referred to as an “action system,” allowing correct focusing of the individual on the environment and providing a coherent behavioral response to external stimuli. The BFCS subserves sensory processing, working memory, attention, learning, and memory by activating ACh receptors in the hippocampus, entorhinal, and frontal cortices (Li et al., 2018; Ballinger et al., 2016). Thus, BFCS disruption may contribute to abnormal development of neuronal circuits in both FXS animal models and patients (Perry et al., 2001; Kesler et al., 2009). FXS is the most common heritable cause of developmental disability, and it is associated with IQ, memory, visuospatial processing deficits, and a profile of cognitive-behavioral deficits predominantly related to executive function, social behavior, communication, and cognitive skills (Reiss and Dant, 2003).

The *fmr1* gene is prominently transcribed during early brain development in the basal forebrain and the hippocampus, two regions critical to memory encoding and attention (Abitbol et al., 1993). More specifically, FMR1-mRNA is expressed with the highest level in BFCN of the Nucleus basalis Magnocellularis (NbM) and in hippocampal pyramidal neurons (Reiss and Dant, 2003; Greicius 2004). The early transcription of *fmr1* gene and brain distribution of FMR1-mRNAs in human fetuses suggest that alterations of *fmr1* gene expression in BFCS may be responsible for FXS typical executive and cognitive dysfunctions (Sarter et al., 2003; Hoeft 2010), and intellectual disability (Abitbol 1993). Furthermore, *fmr1* KO animal models show aberrant cholinergic function in the subiculum and the CA1 region of the hippocampus (D’Antuono et al., 2003), with concurrent behavioral deficits resembling those observed in human FXS patients.

Neuroimaging studies have repeatedly demonstrated abnormalities in prefrontal cortex of individuals with FXS and have shown that lower FMRP is correlated with impaired performance and abnormal prefrontal brain activation during executive tasks (Menon et al., 2004). Males with FXS are at risk for significant cognitive and behavioral deficits, particularly those involving executive prefrontal systems. Reduced grey matter volumes in low functioning autistic children were found in the nucleus accumbens, a BF area critical for adaptive response to aversive stimuli and reward (Riva et al., 2011).

Also, auditory hyper-reactivity is a common sensory-perceptual abnormality in ASD and increases the risk of maladaptive behavior in ASD children. Importantly, BFCS has been implicated in these reflex responses and cholinesterase inhibitors have been proposed to ameliorate auditory sensory response in rodent models (Hohnadel et al., 2007; Jin et al., 2019).

These findings are consistent with a hypothesis of functional disruption of cholinergic systems as a direct consequence of reduced FMRP expression level, resulting in turn in delayed neuronal development and permanent changes in cortical cytoarchitecture, finally leading to cognitive dysfunctions.

Since the etiological causes of ASD remain unknown, the current treatments of this disturbing behavioral profile are largely symptomatic (Mukaetova-Ladinska and Perry, 2015). The use of acetylcholinesterase inhibitors to sustain ach levels, including donepezil, galantamine and rivastigmine, is reported to improve cholinergic synaptic plasticity in rodent models, and executive functions in open label human trials (Ginestet et al., 2007).

Several studies on ASD animal models have shown the involvement of nAChRs in modulating social and repetitive behaviors (Wang et al., 2015). Moreover, CHRNA7 gene mutations have been correlated to autistic-like phenotypes (Yasui et al., 2011). Of note, behavioral abnormalities induced by maternal brain inflammation were prevented by gestational choline supplementation in the offspring of a rodent model (Wu et al., 2015). In line, BFCN have been shown to downregulate brain inflammation *via* stimulation of microglial alpha7 nAChAR (Wang and El-Deiry, 2003; Shytle et al., 2004).

Neuropathological studies conducted on human post-mortem brain tissue of autistic adults confirmed the clinical relevance of both muscarinic and nicotinic acetylcholine receptors in regulating memory and behavioral flexibility deficits in autism. ASD associated changes in α7 nAChAR, as well as muscarinic 1–2 (M1–M2) receptors, have been reported by several groups (Martin-Ruiz et al., 2004; Palma et al., 2012). Further, selective nicotinic and M1–M4 muscarinic agonists lead to enhancement of memory and cognitive performance (Deutsch et al., 2010; Ragozzino et al., 2012; Thomson et al., 2017).

Overall, findings of loss of muscarinic receptors in the brain tissue in autism and FXS and the observed beneficial action of cholinomimetics and Ache inhibitors represent a relevant step forward the development of novel targeted pharmacological treatments for AD, FXS and ASD related disorders.

## FMRP Alterations in Autism and Alzheimer: A Shared Neuropathology?

It is interesting to note that on one hand APP metabolites are considered as potential strategies for FXS, and in turn mGluR5 blocking drugs have been used for AD therapies (Westmark and App, 2019). Two diseases that are characteristic of opposite ends of the human lifespan converge into the same molecular neuropathology. An increasing number of findings highlight the common features between neurodevelopmental issues, on one side, and age-related neurodegeneration, on the other (Triaca and Calissano, 2016; Lahiri et al., 2021), although conclusive evidence is needed.

FMRP is mainly involved in RNA regulation and its absence induces APP overexpression in FXS (Westmark and Malter, 2007; Westmark et al., 2009), as well as in AD (Borreca et al., 2016). Furthermore, and related to FMRP metabolic control at the synapses, the protein synthesis machinery is another common feature found to be altered in both diseases.

These features are only small part of the long story where FMRP plays a key role in two completely different pathologies.

Moreover, a further relevant role for FMRP has also been demonstrated in AD. In fact, fragile X-associated tremor/ataxia syndrome (FXTAS) patients are characterized by an FMRP mutation in 5′UTR of the gene with CGG repetition comprising between 55 and 200, showing a neurocognitive deficit associated with AD (Aydin et al., 2020). Also, other neurodegenerative disorders are associated to FMR1 premutation, such as Parkinson disease or multiple sclerosis (Sacino et al., 2019; Dijkstra et al., 2021), pinpointing a crucial effect of FMRP mutations in common neuronal function and synaptic dysfunction.

Furthermore, FXS and AD share more than molecular substrates, based on compelling evidence on the role of BFCS attention impairment in AD and FXS typical behavioral deficits.

Understanding the molecular link among pathologies affecting brain lifespan from development to ageing is of foremost importance for drug discovery to vanquish pathologies, like FXS, AD, PD, and autism with a deep medical and financial impact on the society.

## Common Future Perspectives

We have highlighted the common features of neurodevelopmental and neurodegenerative disorders. The key molecule, FMRP, is associated to both pathologies and contributes to their phenotypes. How it may contribute to neuronal loss and altered synaptic plasticity in AD is still unknown. This review has described the role of the protein in both pathologies and may offer insight into its double role in neurodevelopmental and neurodegenerative diseases. In more detail, we also described how FMRP has some common features with AD pathology and how this help us to uncover converging mechanisms shared by FXS and other brain disorders. This contributes to the development of a broad-spectrum therapeutic agent.
